# Socially Extended Cognition and Shared Intentionality

**DOI:** 10.3389/fpsyg.2018.00831

**Published:** 2018-05-28

**Authors:** Holger Lyre

**Affiliations:** Department of Philosophy & Center for Behavioral Brain Sciences, Otto-von-Guericke-University Magdeburg, Magdeburg, Germany

**Keywords:** extended cognition, social cognition, shared intentionality, coupling mechanism, cognitive vehicle, mental content, externalism, social content externalism

## Abstract

The paper looks at the intersection of extended cognition and social cognition. The central claim is that the mechanisms of shared intentionality can equally be considered as coupling mechanisms of cognitive extension into the social domain. This claim will be demonstrated by investigating a detailed example of cooperative action, and it will be argued that such cases imply that socially extended cognition is not only about cognitive vehicles, but that content must additionally be taken into account. It is finally outlined how social content externalism can in principle be grounded in socially extended cognition.

## Introduction

Typical examples to illustrate extended cognition include aids such as notebooks, computers, or smartphones, that is, technological artifacts which serve information processing as well as cognitive outsourcing and scaffolding. The possibility, however, that the area of social cognition bears an even bigger potential for cognitive extension has only recently been taken into account. In this paper I want to bring together the topics of *extended cognition* and *social cognition* and then specifically pursue the question in how far the mechanisms of shared intentionality already contain the sources of a social externalism.^1^

I proceed as follows: after this introduction, in the second section, I lay out the claim of extended cognition and discuss some possible misunderstandings. In the third section, I distinguish four domains of cognitive extension, namely extensions into the embodied, the physical, the informational, and the social environment. In order to defend the claim of extended cognition against the charge of cognitive bloat, for each of the areas, and probably even more specifically, one has to find specific conditions and mechanisms of cognitive coupling to external components. In the fourth section, I set the claim of extended cognition, a claim about cognitive vehicles, off from an externalist claim about content. In accordance with Clark and Chalmers, I characterize the latter as “active externalism” and illustrate its differences and unique features vis-à-vis the familiar passive forms of semantic or mental externalism in the philosophy of mind commonly associated with names such as Putnam, Millikan, and Burge. In the fifth section I follow up on the specific idea of considering the mechanisms of shared intentionality as coupling mechanisms of cognitive extension into the social environment, in particular, partners in social interaction. In so doing, I make detailed use of Bratman’s well-known planning theory of shared intentionality. On the basis of the results of this analysis, we will then in the sixth and final section see in which sense we are here already dealing with the seed-bed of a form of social externalism. Hence, social externalism turns out to already represent a precursory version of active externalism.

## Extended Cognition as a Claim About Vehicles

Cognitive systems and subjects spread out beyond their traditionally assumed physical boundaries into the world, they are not merely situated in the world, but inherently world-bound – this is the basic idea of the by now classic 1998 “Extended mind”-paper by Andy Clark and David Chalmers.^2^ From 2000 onward, however, Clark, the key proponent of the claim, has increasingly and preferably spoken of “extended cognition.” Why this change of term? It obviously has to do with Clark having wanted to stress that he is concerned with a claim about cognitive vehicles, that is, processes, activities and states of the cognitive machinery and not with a claim about cognitive content. Particularly the work of Hurley (1998, 2010) has obviously been influential. In Clark’s own words (Clark, 2005, Fn.1): “*It is important... to maintain a distinction between vehicles and contents. Possessing a contentful mental state is most plausibly a property of a whole active system (perhaps in some historical and/or environmental context). Within that system, certain enduring material aspects may play a special role in enabling the system to possess (whether occurrently or dispositionally) a given mental state. These material aspects are the vehicle of the content. The Extended Mind hypothesis is really a hypothesis about extended vehicles, vehicles that may be distributed across brain, body, and world. We conflate vehicles and contents, as Dennett (1991) and Hurley (1998) stress, at our philosophical and scientific peril.*”

Surely, advocates of the idea of cognitively extended systems must likewise be able to explicate what “non-extended” cognitive systems are. Traditionally the brain (or, more generally, the central nervous system) is seen as the vehicle of cognition. Hence, proponents of the claim that cognitive states, processes, and mechanisms go beyond states, processes, and mechanisms of the brain are advocating a variant of extended cognition. Take as an example the activity of doing a jigsaw puzzle. It proves to be helpful to pick up pieces of the puzzle, turn them around in one’s hand and thus try out to solve the cognitive task in direct embodied interaction with the jigsaw puzzle. On the traditional view, embodied actions are not part of the “actual” cognitive goings-on which are restricted to brain processes such as the perception of the mental rotation of puzzle pieces and the evaluation of shape fit. From here one arrives at extended cognition in two steps. In order to master the task posed by the jigsaw puzzle, the brain could draw both on sensory and motor formats of representation which – in the sense of Gibsonian “affordances” – correspond to the operations of grasping and turning enabled by the hands. This would be one possibility of interpreting the idea of embodied cognition: internal representations are afforded and shaped by embodied and situational opportunities. Proponents of extended cognition take things one step further and claim that the “epistemic actions” (cf. Kirsh and Maglio, 1994) of doing jigsaw puzzles do not merely serve to outsource internal computation, but are an integral part of the entire cognitive goings-on and cannot, as such, be severed from it in a natural manner. The cognitive loop encompasses states, processes, and mechanisms both of the brain and the body as well as of the environment (in this case the jigsaw puzzle). The set of cognitive vehicles, as against the brain, is extended.

## Four Domains of Extended Cognition and Their Mechanisms of Coupling

The example of the jigsaw puzzle shows that extended cognition inevitably entails the claim that the external components (external states, processes, and mechanisms) serving cognitive extension are temporally highly variable – depending on the cognitive task posed. So cognitive systems or subjects, insofar as they are individuated by means of their physical vehicles or realizers, become fluid and variable as well. But why stop at the movement of the hands of the person doing the jigsaw puzzle? Why not count her vital functions as part of the cognitive loop? Shouldn’t we think of the mental states of any Internet user as spread out over the whole web? This is the well-known objection of “rampant extension” or “cognitive bloat.” In order to avoid such objections, specific criteria need to be provided under which alone it is justified to count certain external components as extensions of the original system. C&C have already formulated three general requirements that external components have to satisfy: first, direct *accessibility* at any point in time of the task, second, stability and r*obustness* on the relevant time scale as well as, third, reliability or *validity*.

Accessibility, robustness, and validity provide general and gradual criteria. What degree of, say, accessibility of an external component is needed in order to be sufficient for cognitive extension? As a rule of thumb and we may say that the degree of accessibility, robustness, and validity should be comparable to the degree of accessibility, robustness, and validity of comparable internal components (e.g., the neural vehicles of internal biological memory compared to external memory tools). This is in the spirit of C&C’s parity principle, the claim that if a part of the world is functionally equivalent to a part of a process done in the head, then that part of the world is part of the cognitive process (Clark and Chalmers, 1998, 8; cf. Clark, 2008, 77ff.). But while accessibility, robustness, and validity give us general criteria of the coupling, they do not specify the various possible coupling mechanisms. It is important to note that such coupling mechanisms may drastically vary, since cases of cognitive extension may occur in rather different domains. I propose to distinguish four primary domains of cognitive extension:

(1) The body
(2) The physical environment
(3) The “informational” environment
(4) The social environment

I suggest that, as long as we are dealing within the context of cognitive vehicles, the criteria of extension are *purely functional criteria of causal coupling or mechanistic integration* (a claim, again, in the spirit of C&C). Consider the following examples. The jigsaw puzzle or the oft-cited example of Tetris (another example of epistemic actions in the sense of Kirsh and Maglio, 1994 as used in Clark and Chalmers, 1998) both involve cognitive extensions into the bodily and physical environment. Very often, in such cases, precisely those elements of the physical environment are implicated in which the cognitive system, in virtue of highly dynamic sensory-motor feedback loops, is not only embedded and situated but with which it is inextricably coupled. The cognitive loop of a table tennis player, for instance, comprises certain afferent and re-afferent perceptual states, efferent embodied and movement states as well as states of the ball, the bat, the table, the air pressure, etc. In a similar manner a blind person’s cane, glasses, or prostheses enter into the extended cognitive (here specifically sensory-motor) loop. So in domain (1), one finds coupling mechanisms which correspond to those mechanisms that are examined and identified in the fields of embodied and situated cognition, especially mechanisms of proprioception. Domain (2), extension into the physical environment, comprises mechanisms and stylistic principles of dynamicism, especially sensory-motor feedback coupling under inclusion of the principle of reafference.

The examples illustrate the strictly *functionalist strategy*. An external vehicle is considered sufficiently coupled if the connections to the original system and the bandwidth of coupling *have achieved approximately the degree that the parts and components of the original system, too, have amongst themselves.* The extended system, which is indeed the legitimate cognitive system, thus forms an organizational unit with regard to its vehicles due to its sufficient degree of mechanistic integration [see Menary (2007) on cognitive integration and Zednik (2011) on extended cognitive mechanisms]. To put it with a slogan: *It’s all about bandwidth and coupling.*^3^ The functionalist strategy is the one most appropriate for extended cognition; for this doctrine is primarily a functionalist doctrine: it’s all about achieving cognitive tasks and functions, the nature of the vehicles is of secondary interest only. Certain examples of extended cognition may therefore remind us of examples of so-called “strange realizations” from the early debate on functionalism. But if the “extended puzzle player” arrives at a satisfactory solution of the cognitive task, there are no functionalist reasons to not see the extended puzzle player as a legitimate cognitive-systemic whole. Proponents of extended cognition conversely argue that brain-bound accounts of cognition fall prey to an unjustified neuro-chauvinism.^4^

Let us now turn to examples of domain (3): extension into the “informational” environment. Characteristic examples are the already mentioned tools such as notebooks, computers, or smartphones. They are that part of the physical, typically culturally shaped environment which serves to process, store or otherwise manipulate informational elements or meaningful symbols. The crucial basis for most of this is a structured and symbolically encoded language (albeit, of course, domain (3) might include non-symbolic entities as for instance drawings or music as well). It ultimately allows for the powerful possibility of externalizing the content of cognitive acts, of making them public and sharing them with others – undoubtedly a decisive step in the cognitive arms-race of more recent human evolution. On a commonly held view, this step serves to “externalize” what is already present internally in a cognitive creature. The structure of a public language just follows the prior structure of the “Language of Thought” (Fodor, 1975). But from the point of view of extended cognition, the order of arguments should be inverted. It is the possibility of actively structuring the physical environment afforded by the world that significantly drives the development of symbolically structured and linguistically encoded thought.

The integral coupling of a seemingly purely external symbolic language is all too easily overlooked in everyday life – unlike, however, where it serves feats of intellectual brilliance. Clark reports an encounter between Charles Weiner, a historian, and Richard Feynman. In conversation Weiner acknowledged Feynman’s notes and sketches as historical documents of his work: “[b]ut instead of simply acknowledging this historic value, Feynman reacted with unexpected sharpness: ‘I actually did the work on the paper,’ he said. ‘Well,’ Weiner said, ‘the work was done in your head, but the record of it is still here.’ ‘No, it’s not a record, not really. It’s working. You have to work on paper and this is the paper, okay?”’ (Clark, 2008, p. XXV). Indeed, Feynman’s formulation of quantum electrodynamics or Einstein’s derivation of the field equations of the general theory of relativity would be inconceivable without pen, paper, and mathematical symbolism. Feynman stresses how much the activity of a creative mathematical physicist is dependent on this fact. One could see this as an acknowledgment of the external tools of his cognitive work, but it seems even more appropriate to say that a productive scientist of Feynman’s caliber has a fine sensitivity for how much pen, paper, and mathematical symbolism are an integral part of his cognitive capacity.

Another notorious example for domain (3) is C&C’s Alzheimer’s-patient Otto. As a paradigmatic example for many amnesiacs, Otto uses a notebook as his constant companion, into which he enters all relevant information that he needs in everyday life. From the point of view of extended cognition, the notebook is his extended memory, for he continuously consults it, fully relying on the reliability and authenticity of its entries – in the same manner in which we all access the phone numbers of friends and relatives with a few clicks on our smartphones instead of keeping them in mind.

The new and remarkable point here is that we are not only talking about fast and robust accessibility of the notebook or smartphone, but that in order to justify the information contained in an external memory component as valid, we need criteria of cognitive ownership and conceptual integration (cf. Kyselo and Walter, 2011). Such criteria, however, essentially aim at meaning, they are about content, not (just) about vehicles. What is meant by “cognitive ownership” requires a thorough analysis, but the basic idea is easily illustrated. The entries in Otto’s notebook must have a certain degree of consistency and coherence with the remainder of his belief system, more precisely put: it must be possible to “somewhat” consistently and coherently integrate them into this system in order to become part of the extended belief system which can then be ascribed to Otto-plus-notebook. If, for example, Inga is Otto’s best friend with whom he gets on very well, then an entry such as “I am always fighting with Inga” makes little sense. There will not be many such inconsistencies, since Otto makes the entries into his notebook himself. But it is also conceivable and not contradictory if Otto, on occasion, entrusts someone with the authority to make an entry for him. However, in all cases errors can occur. The entries only have to be “somewhat” consistent, since none of us has an ideally rational belief system. In this respect, the criterion of cognitive ownership follows that of the mechanistic coupling of vehicles: the degree of integration in semantic terms only has to be as good as that of the various component beliefs of ordinary belief systems.

Critics have also pointed out that, as against biological memory, the notebook is much more prone to errors and manipulation, but here, too, what matters is weighing things gradually. By no means is our memory always reliable and is not immune to manipulation. Still, several questions concerning this matter are still open and it remains a desideratum of the debate on extended cognition to formulate content-based criteria for domain (3) such as cognitive ownership and conceptual integration in more detail.

Finally and before we go on, some general remarks on the connections and possible dependencies of the four domains. It could be argued that embodiment, i.e., the domain-(1)-extension into the body, is a prerequisite of the three other domain-extensions. It could further be argued that the informational and the social environment are sub-types of the physical environment.^5^ But while all this seems to be true for the majority of cases, it’s not necessarily true. Of course: how else should a cognitive system be coupled to the physical, informational, or social environment if not via its body? And isn’t it true that all informational tools and social partners must somehow be physically realized? But consider a neural implant that in some future may be able to directly enhance my mathematical abilities. It provides an example of domain-(3)-extension into the informational environment without relying on the body or the physical environment (but rather on the brain). Or, to become even more futurological, such an implant could even impersonate an AI system in its own right that is now directly coupled to my brain. An instant and inside cooperating partner, as it were. My hunch would be that this now counts as domain-(4)-extension rather than (3). But borderline cases may also exist. The domains need not to be clear-cut, and their more fine-grained distinction provides future work. Finally, and as we shall see in the next two sections, a characteristic feature of domains (3) and (4) as compared to (1) and (2) is that the vehicle-content distinction becomes relevant. I shall argue that, rather than external components of the bodily and physical domains, components of the informational and social domains can typically be individuated both on the vehicle and the content level. For the domain-(3)-extension into the informational environment this was already indicated in our discussion of the Otto-Inga case. As we shall see in Section “Shared Intentionality as the Coupling Mechanism of Social-cognitive Extension,” matters are similar for domain (4), extension into the social environment. This clearly distinguishes (3) and (4) from (1) and (2).

## From Vehicle- to Content-Externalism

Before we scrutinize the relevance of coupling criteria both on the level of vehicles and of content, it is perhaps worth noting that our distinction between vehicle and content can be understood in a purely conceptual and methodical manner and does not necessarily commit us to any sort of content realism. It is perfectly possible to re-conceive all ideas put forward here in the frame of content instrumentalism according to which talk of meaning, semantics and mental content is explanatorily useful, maybe even relevant, for cognitive science and philosophy of mind without entailing any stronger ontological commitments.

Let us begin with the widely held assumption that mental content supervenes on cognitive vehicles. For internalists this requirement comes quite naturally: mental content – be it pre-conceptual, conceptual, or propositional content – supervenes on brain states. In this sense, mental content depends on vehicles, but is multiply realizable by various vehicle tokens. Rowlands (2003, 13) has pointed out that (Cartesian) internalism should be understood as a combination of two claims: a *location claim*, according to which mental phenomena are located inside the spatio-temporal boundaries of a (brain-bound) cognitive subject or system S, and a *possession claim*, according to which mental phenomena do not depend on factors or features external to the bounds of S. While the location claim is directly applicable to the vehicles of mental phenomena, the possession claim more aptly applies to content. This, yet again, emphasizes the ontological neutrality of talk of content: mental contents are ascribed to vehicles, they need not necessarily be ontologically charged. It follows that internalists assume that mental content is intrinsic to S, i.e., independent of external factors, while externalists construe mental content as relational. As we have seen, the claim of extended cognition is at first a claim about the possibility of extending the set of vehicles. Due to the content-vehicle supervenience it is, however, straightforward to assume that in cases of extended cognition mental content should be ascribed to the entire extended system.

Consider the following example: when asked whether they know what the time is most people react with an explicit ‘yes,’ look at their watch (or smartphone) only afterwards, and finally give a response. One may discard this as mere elliptical speech, but it likewise indicates how self-evident and natural it has become for us to wear a watch as a constant personal companion. If we take the statement literally, then people attribute to themselves a knowledge state which they couldn’t attribute without the watch. This entails two things: first, the extended system S-plus-watch seems to be the legitimate cognitive system, hence S→S^∗^ = S+E (where S refers to the original, non-extended system and E to a particular external component). Second, it is the extended system S^∗^ that ‘knows the time.’ This is the system to which the knowledge state should be ascribed.

Consider the following analogous case: if Otto wants to meet Inga at the Museum of Modern Art, he looks up the address in his notebook. We should then ascribe the knowledge of the MoMA’s address to the extended system Otto^∗^ = Otto + notebook. So by no means is it the case that extended cognition leads to absurd claims about knowledge states of notebooks and the like. Since Otto has a (standing) belief that is in part constituted by his notebook, the claim of extended cognition correctly asserts that the extended vehicle system Otto^∗^ has beliefs. In this sense, extended cognition in conjunction with content-vehicle supervenience leads to a form of “extended internalism” (with beliefs internal to the extended system Otto^∗^, for instance). Curiously, however, C&C have used the label “active externalism” instead. In Lyre (2016) I have argued that both labels should be considered synonymous as far as vehicles are concerned, but that extended cognition – in domains (3) and (4) – does indeed lead to a new and remarkable sort of content externalism for which the label active externalism is more fitting. Let us have a closer look at this.

In their 1998 paper, C&C in fact neglect the content-vehicle distinction (which has led Clark to self-criticism; see footnote 1). Nonetheless they extend the Otto example to accommodate Twin Earth cases, as is common in debates on content externalism. In the sense of a possession claim, content externalism means that mental content depends on external factors or components. Three forms are generally distinguished, known as *physical, historical*, and *social externalism.* They can be related to different (groups of) theories of meaning: causal theories of reference, teleosemantics, and use theories of meaning. According to the causal theory of reference, meaning depends on the nature of reference objects to which we are linked by some sort of causal chain. This implies externalism: mental content depends on external natures and not just on the internal states of Putnam’s (1975) notorious Twin Earth thought experiment illustrates the consequences: Oscar’s thoughts about water depend on the nature of water, i.e., H_2_O. Twin Earth is an exact physical duplicate of Earth with the only exception that the external, content-fixing component, in this case the nature of water, is different. On Twin Earth water is XYZ and, as such, the content of Twoscar’s thoughts about water is different from the content of Oscar’s thoughts about water on Earth. Strangely enough, neither Oscar nor Twoscar must be aware of this difference, nor is it in any way relevant for their behavior. The analog holds true for a corresponding variation in the teleos history of an expression on Twin Earth in the context of historical externalism or for a change in word usage within a linguistic community in the context of social externalism. Traditional forms of externalism can thus aptly be characterized as “passive externalisms”: a cognitive system has no influence on the external, content-fixing components, nor do these components causally influence (the vehicles of) S.

In a similar manner, C&C consider the Alzheimer’s patient Twotto on Twin Earth who wants to meet Twinga, but who finds the 51st Street as the address in his notebook. Due to this erroneous entry – a variation of an external component of the extended system Twotto-plus-notebook – there is a behaviorally relevant change: Twotto’s appointment with Inga fails. Hence, C&C’s talk of active rather than passive externalism. In large parts of the literature, the label active externalism has become a synonym for vehicle externalism (or even extended cognition in general). But the example only works in virtue of content, that is, in virtue of the behaviorally relevant knowledge states of the extended subjects of knowledge: Otto^∗^ and Twotto^∗^. It thus makes perfect sense to see the label as a new brand of content externalism – a variant that is implied by the vehicle-extension in conjunction with content-vehicle-supervenience. Whether we want to alternatively speak of an *(active) extended content internalism*, as already mentioned, is mostly a verbal point. In the latter case, however, the intuition would be lost that cognitive systems, from the point of view of extended cognition, are temporally fluid and variable with regard to their vehicles. In an overwhelming amount of cases the cerebral vehicle, the brain, can be seen as the cognitive core system the extra-cerebral, external components of which vary.^6^ Thus, the term *active (content) externalism* seems fitting.^7^

## Shared Intentionality as the Coupling Mechanism of Social-Cognitive Extension

We now turn to domain (4): extension into the social environment. Not only artificial external components can serve informational out-sourcing, but also partners in social interaction. This can occasionally be seen in long-standing couples. One partner, due to their own fading abilities, relies on the memory of the other. Thus the latter takes on the role of Otto’s notebook or an extended memory, respectively. Sutton et al. (2010) provide a rich and detailed empirical as well as conceptual analysis of such cases which they situate within a multidimensional framework spanning the border between distributed (in the sense of Hutchins, 1995) and “scaffolded” social cognition (in the sense of Sterelny, 2010) on the one hand and socially extended cognition on the other. Kosslyn (2006) speaks of “social prosthetic systems,” where “other people serve as prosthetic devices, filling in for lacks in an individual’s cognitive or emotional abilities.”

Among the conditions and mechanisms of coupling in the domain of social extension we can quite naturally count spoken language. But language is only one such mechanism, albeit a highly developed one, as coupling mechanisms in this domain are numerous and diverse. One can make the general claim that virtually all mechanisms studied in *social cognition*, from the point of view of extended cognition, can be seen as potential coupling mechanisms of social extension. This leads to an impressive list of candidate mechanisms:

• following movement trajectories
• behavior reading
• eye-tracking
• joint attention
• body posture
• gesture
• facial expression
• shared goals
• shared (also collective or we-) intentionality
• co-operative action
• communicative action
• mind reading, mentalizing, theory of mind
• social learning
• group-based norms
• language
• social and cultural institutions

The list could be continued or more finely differentiated (especially toward the end). Each of these topics would deserve a special analysis from the perspective of extended cognition. Krueger (2011), for instance, has discussed some of the basic mechanisms, in particular gesture, body posture, and facial expression, in the context of extended cognition and argues for seeing these mechanisms as an interactive form of shared action-space management of embodied agents (“we-space” management). Borghi et al. (2013) consider words as social tools of the embodied-grounded and extended mind. And Gallagher contemplates on “the socially extended mind [that] is in some cases constituted not only in social interactions with others, but also in ways that involve institutional structures, norms, and practices” (Gallagher, 2013, p. 4).^8^

Our focus should lie on *shared intentionality*, arguably the most important and most-studied social-cognitive mechanism of the last two decades (cf. Schweikard and Schmid, 2013; Jankovic and Ludwig, 2017). Tomasello has long argued that cooperative social interaction is even the key to our cognitive uniqueness (cf. Tomasello, 2008, 2014). Metzinger and Gallese (2003) show in particular that the brain aims at modeling the physical and social world in terms of a distinct ontology of shared actions and goals. And in reviewing the connections between shared intentionality and extended cognition, Gallotti and Huebner (2017) muse about the possibility that the range of mental contents and operations can indeed be extended through shared actions. What is still missing, however, is a more detailed analysis of the coupling mechanisms for socially extended cognition and how such an analysis contributes to the distinction between vehicles and contents and the corresponding distinction between vehicle externalism and content externalism.

To enter into this, let’s consider a particular example. Assume Cindy and Bert intend to jointly publish a book. In so doing, they divide the responsibilities amongst each other. Cindy could, for instance, be responsible for typesetting and layout, while Bert proof-reads. Neither of them needs to know in detail how the other fulfills their part, they do, however, co-ordinate their timing. If Bert, on occasion, isn’t sure about how orthography works in a particular case, then Cindy, who also knows her grammar, will gladly help him. The example satisfies the features of co-operative action as put forward by Bratman (1993) in the context of his well-known *planning theory of shared intentionality*: (1) mutual co-ordination, (2) commitment to joint action, and (3) commitment to mutual support. Let us here assume that an action is founded on an intention and a plan to act. Cases of shared agency are thus also cases of shared intentionality. In the case of co-operative action, the action may consist of sub-plans which are different for each partner in co-operation and which mesh – this is of the essence for both Bratman’s analysis and our question.

Cindy and Bert know for themselves that they are publishing a book with the other. With a view to the overarching goal each of them individually knows how to do it. But not every agent plans or knows all details und sub-plans of the shared action. Cindy determines font size, Bert decides matters of punctuation and doesn’t care about font size. While the overarching action plan can be reduced to the partial plans of the agents, it cannot be ascribed to one of the agents individually. This is in line with Bratman’s individualistic and reductionist conception: a co-operative action can be traced back to the individual partners in co-operation. According to Bratman, one can thus intend to jointly J, but not *jointly intend* to J (J stands for a joint-act-type). Thus Bratman guards against the ideas of a collective agent or group mind the intentional states of which are causally efficacious for the collective. This is in line with extended cognition, for here, too, the aim is not to fuse different cognitive systems into meta-systems (contrary to, for instance, Tollefsen, 2006), but to flexibly and in a task-sensitive manner extend the boundaries of individual systems. According to Bratman (1993, p. 106), the specific conditions of co-operative action, which our example satisfies, are:

“We intend to J if and only if

(1) (a) I intend that we J and (b) you intend that we J.
(2) I intend that we J in accordance with and because of la, lb, and meshing subplans of la and lb; you intend that we J in accordance with and because of la, lb, and meshing subplans of la and lb.
(3) 1 and 2 are common knowledge between us.”      (^∗^)

Several objections have been made to Bratman’s influential analysis, but this need not particularly concern us here. Bratman has reacted to some of these objections over time and his most recent conception (Bratman, 2014) contains some improvements, but has not changed at its core with a view to the planning theory.^9^ It is precisely this core idea that is of particular interest for us. We can also leave aside the possibly justified objection that Bratman’s conception is cognitively too demanding, because it presupposes extensive mind-reading abilities (and thus, for example, excludes infants, children, and apes). But even if that means that Bratman’s conception should be preceded by a cognitively less demanding conception, we can nonetheless see it as a plausible candidate and thus *pars pro toto* for the conception of shared intentionality among adults.

If one now takes the perspective of extended cognition, Bratman’s conditions (^∗^) can not only be seen as conditions of shared intentionality, but equally as coupling conditions for the social extension of a subject into processes and states of another socially embedded subject. For in cases of shared intentionality a particularly strong coupling with partners in co-operation is required; this has to take a form that the sub-plans and sub-intentions mesh and interlock. Precisely this makes Bratman’s planning conception so interesting from the point of view of extended cognition. In this sense, I claim that *shared intentionality is a candidate mechanism of domain (4) with coupling conditions provided by (^∗^)*.

This means that those cognitive states and processes on which the co-operative actions of Cindy (or Bert resp.) are based partly also include cognitive states and processes of Bert’s (or Cindy’s resp.). So the cognitive loop contains vehicles of the other, a case of socially extended cognition. These are each those states or processes that in conditions (^∗^) serve to provide contributions of the other. Since we are here dealing with intentions and shared knowledge, in particular, the sub-plans and sub-intentions of the partner(s), the coupling conditions in the special case of shared intentionality, as in other cases belonging to domain (4) such as the long-standing couple, are related not to vehicles but to content similar to domain (3).

This circumstance brings up the question of whether in cases of shared intentionality we are not just dealing with a form of extension of cognitive vehicles, but whether this, as in the case of Otto’s notebook, entails a form of content externalism. I want to argue that the answer is a “restricted yes,” that it is not a pure content externalism, but an interesting precursor of it. Here we need to go deeper into our example. Which intentional states or belief contents can be ascribed to Cindy and Bert? Cindy believes that she and Bert are co-operating. Let the content of her corresponding belief state be I(C). In like manner, Bert believes to be co-operating with Cindy with belief content I(B). It could clearly be the case that Cindy and Bert each individually and independently are in the mentioned belief states, for instance, if they are mistaken about the good intentions of the other. This then would not be a case of shared intentionality and thus *a fortiori* also not a case of social cognitive extension.

But if Cindy and Bert satisfy conditions (^∗^) with regard to co-operation, it is not the original subjects Cindy and Bert which have the corresponding belief states, but the socially extended subjects Cindy^∗^ and Bert^∗^. For the contents I(C^∗^) and I(B^∗^) of their mutual beliefs “We are co-operating” it holds true that I(C^∗^) = I(B^∗^). Now our example has been designed such that I(C) = I(B) holds true, even I(C) = I(B) = I(C^∗^) = I(B^∗^). This shows that it cannot (always) be detected at the level of the belief contents whether one is dealing with a case of shared intentionality (or cognitive extension, respectively). However, it is necessarily true that I(C^∗^) = I(B^∗^) in case we are dealing with a real case of shared intentionality, while I(C) = I(B) is merely contingently fulfilled (as, for instance, in our example). Furthermore, the (self-reflexive) beliefs of Cindy^∗^ and Bert^∗^ with the contents I(C^∗^) and I(B^∗^) are necessarily true, while the beliefs I(C) and I(B) of Cindy and Bert can be either true or false.

Insofar as the beliefs I(C^∗^) and I(B^∗^) supervene on the extended cognitive vehicles of Cindy^∗^ and Bert^∗^, we are dealing with a form of content externalism that is fully analogous to Otto’s MoMA address knowledge. As the example shows, one cannot detect this at the level of contents, but at the level of their supervenience bases only. Cognitive intentional acts in conjunction with co-operative action are noteworthy externalist borderline cases. But are we, as in the case of Otto’s notebook dealing with a case of active externalism? A simple consideration shows that this is the case. In active externalism, changes in the external component are behaviorally relevant. Cindy’s belief that over the course of the next 10 months she will be working on the publication of a book changes and thus also her behavior if Bert doesn’t fulfill his part of the co-operation in the time originally planned, but by changing some of his sub-plans, causes the joint book project to last 12 months. Shared intentionality mutually depends on both partners in co-operation and both condition and influence one another actively.

## Outlook: Social Externalism as Active Externalism

Language is obviously a very important cognitive vehicle. Tomasello (2008, 2014) has contributed significantly to the idea that language and linguistic meaning have their origin in shared intentionality. In the context of a use theory of meaning, toward which Tomasello, too, has leanings, one is led to a social content externalism. The reason for this is that, on a use-theoretic view, linguistic meanings supervene on their functional roles in the linguistic community. This implies a social externalism according to which the semantic content of mental states is determined by circumstances in the social environment of a cognitive subject, in this case, the functional roles of linguistic usage.^10^

Social externalism, like physical and historical externalism, too, is typically construed as a passive externalism. On the other hand, we have seen that a precursory form of social externalism occurs in the mechanism of shared intentionality which is active in nature. *Prima facie*, there is a tension here. But this tension can be resolved, for on closer inspection there are hints that social externalism, strictly speaking, is an active externalism, too.

Proponents of social externalism such as the late Wittgenstein (1953; see also Child, 2006) or Tyler Burge consider to what extent the meanings of linguistic expressions depend on the usage in a linguistic community. A paradigmatic Twin Earth scenario by Burge (1979) is generally well known: Oscar thinks that arthritis is not just an inflammation of the joints but also of the bones and thus has the false belief that he has arthritis in his thigh. On Twin Earth, however, in which the word “arthritis” comprises inflammations of the bones, too, Twoscar has the true belief that he has “arthritis” in his thigh. Due to the usual division of linguistic labor, Oscar only becomes aware of his erroneous usage when in contact with an expert, for instance, when visiting a doctor.

The thought experiment bears out the point that linguistic meanings depend on the patterns of use in the community. Oscar has no influence on how the expression “arthritis” is used in English, especially not among experts. But has he really got no influence in principle? By use of a small thought experiment, I have attempted to argue that Oscar does indeed have an influence, but that this influence is *de facto* negligible due to the sheer size of the language community (Lyre, 2016). Language is typically manifested in large communities – this is what the passive element of social externalism turns on. But in borderline cases from smaller linguistic communities we can, however, increasingly see the possibility of each individual speaker influencing the linguistic usage in the community. So in such borderline cases there is a transition from passive to active social externalism. Strictly speaking, social externalism proves to be a disguised active externalism which is, however, for all practical cases, a passive externalism. Thus social externalism fits very well into the conception of socially extended cognition. It in fact helps to shed light on how extended cognition contributes not only to vehicle externalism but to an interesting form of content externalism as well.

## Author Contributions

The author confirms being the sole contributor of this work and approved it for publication.

## Conflict of Interest Statement

The author declares that the research was conducted in the absence of any commercial or financial relationships that could be construed as a potential conflict of interest.

## Footnotes

1 There is in fact a growing literature that discusses connections between topics from social cognition and ideas from embodied, situated, and distributed cognition as well as enactivism. References of interest include Metzinger and Gallese (2003), Tollefsen (2006), De Jaegher et al. (2010), Sutton et al. (2010), Krueger (2011), Borghi et al. (2013), Gallagher (2013) and Gallotti and Huebner (2017). Our special focus will be on the connection between extended cognition and “shared intentionality" (broadly construed the view that intentions, goals and actions might be shared among cooperating individuals). Papers that particularly relate to our special focus will explicitly be addressed in the beginning of Section “Shared Intentionality as the Coupling Mechanism of Social-cognitive Extension".

2 The joint authors Clark and Chalmers will henceforth be referred to as C&C, and their paper as Clark and Chalmers (1998) resp.

3 For precisely this reason one should agree with Clark (2009) that the claim of extension is not plausibly to be applied to phenomenal consciousness. While, due to the notorious explanatory gap, there is no agreement on the physical mechanisms and vehicles of qualia, our contemporary knowledge of neuroscience suggests that, in order to bring about consciousness, highly integrative and dynamic mechanisms with great bandwidth over short temporal scales are required, as could (presently) only arise within the neuronal machinery.

4 In a corresponding manner one should deal with the coupling-constitution-fallacy made prominent by Adams and Aizawa (2008). The functionalist needn’t acknowledge constitutive criteria of cognition beyond sufficient causal coupling. Furthermore, the doctrine of functionalism is blind to the question of what the mark of the cognitive is. Here, anti-functionalists remain notoriously unsatisfied. But this is not a specific weakness of the claim of extended cognition; at best, of functionalism or, perhaps more plausibly, of its opponents who are chasing a ghost.

5 Thanks to an anonymous reviewer for raising both points.

6 This need not be understood as a strong commitment to a cognitive core system, about the existence of which there is considerable debate among proponents of extended cognition.

7 Of course, not every variation of external components leads to a change in mental content. Consider a case in which Otto has a back-up copy of his notebook (being his extended memory, after all!); in that case it wouldn’t matter in which of the two notebooks he looks up the address of the MoMA.

8 See also the related papers in the special issue on “Socially Extended Cognition” in *Cognitive Systems Research* 25–26 (2013), pp. 1–72.

9 Cf. Schweikard and Schmid (2013) as well as a book symposium on Bratman (2014) in Journal of Social Ontology 1(1) 2015.

10 For reasons of space, I here leave out the distinction between linguistic meaning and mental content. On their relation and connection with an (internal + external!) functional role semantics see Lyre (2016).
